# Finite Element Analysis and Experimental Validation of the Anterior Cruciate Ligament and Implications for the Injury Mechanism

**DOI:** 10.3390/bioengineering9100590

**Published:** 2022-10-21

**Authors:** Shuang Ren, Huijuan Shi, Zhenlong Liu, Jiahao Zhang, Hanjun Li, Hongshi Huang, Yingfang Ao

**Affiliations:** 1 Beijing Key Laboratory of Sports Injuries, Department of Sports Medicine, Institute of Sports Medicine of Peking University, Peking University Third Hospital, Beijing 100191, China; 2 Biomechanics Laboratory, College of Human Movement Science, Beijing Sport University, Beijing 100084, China

**Keywords:** anterior cruciate ligament, finite element method, stress, knee

## Abstract

This study aimed to establish a finite element model that vividly reflected the anterior cruciate ligament (ACL) geometry and investigated the ACL stress distribution under different loading conditions. The ACL’s three-dimensional finite element model was based on a human cadaveric knee. Simulations of three loading conditions (134 N anterior tibial load, 5 Nm external tibial torque, 5 Nm internal tibial torque) on the knee model were performed. Experiments were performed on a knee specimen using a robotic universal force/moment sensor testing system to validate the model. The simulation results of the established model were in good agreement with the experimental results. Under the anterior tibial load, the highest maximal principal stresses (14.884 MPa) were localized at the femoral insertion of the ACL. Under the external and internal tibial torque, the highest maximal principal stresses (0.815 MPa and 0.933 MPa, respectively) were mainly concentrated in the mid-substance of the ACL and near the tibial insertion site, respectively. Combining the location of maximum stress and the location of common clinical ACL rupture, the most dangerous load during ACL injury may be the anterior tibial load. ACL injuries were more frequently loaded by external tibial than internal tibial torque.

## 1. Introduction

The anterior cruciate ligament (ACL) plays a crucial role during daily life and sports activities, as it resists forces on the knee joint to maintain knee stability. However, the ACL is very susceptible to injury and is one of the most common sports injuries. ACL injuries usually have severe effects on the human body [1], including reduced knee stability, secondary injuries to the meniscus and long-term disability [2,3,4]. More than half of all ACL injuries are non-contact, usually more preventative [5,6]. Understanding the mechanism of injury is the key to the effective prevention of ACL injuries.

A large number of experimental works have been performed to investigate the ACL injury mechanisms, the main methods of those studies being the video analysis of sports footage [7,8], simulations of ligamentous injury using cadavers [9,10] and the analysis of dangerous movements in the laboratory [11,12,13]. Recently, biplanar radiography has been used to investigate in vivo ACL deformation under various loading conditions. Previous studies reported that anteromedial and posterolateral ACL bundles were maximally elongated near knee extension [14]. Englander et al. further investigated in vivo ACL deformation during dynamic movement and found that peak ACL strain occurs at an extended knee position [15]. Although these studies have greatly helped us understand knee biomechanics and injury mechanisms, these methods cannot observe the stress of the ligament itself. The stress distribution of the ACL is critical for understanding its mechanical behavior and injury mechanisms. The finite element (FE) method could provide a new perspective for understanding the mechanical behavior of biological tissues. The FE method has also become a powerful tool for calculating the local cyclic stresses and strains through bending and torsion fatigue specimens [16]. The FE procedure is also a suitable method for calculating the cumulative damage and predicting fatigue damage accumulation [17]. Establishing the FE model to simulate injury-prone actions in order to obtain biomechanically relevant properties of ligaments is also a meaningful direction.

In addition to experimental studies, many FE models of the knee joint have been used to investigate ACL biomechanics. A common method in previous FE studies was to model the ligaments as springs [18,19,20]. Li et al. developed a knee FE model that was validated using a cadaveric specimen [19]. In their model, ligaments were modeled using nonlinear springs. Homyk et al. [20] investigated what loads could increase ACL loading based on the established knee model by setting them as multi-stranded nonlinear spring units. The ligaments modeled with spring elements may help investigate knee joint kinematics. However, the stress distribution of the tissue is not available based on spring elements. Some other researchers developed a validated knee model by modeling the ligaments as the geometric structure to investigate the ACL stress distribution [21,22,23,24,25]. In these studies, some models did not include all the critical anatomic structures of the knee joint, which are complex and essential for exploring joint function. Moreover, the accuracy of the ACL geometric characteristics of the existing knee FE models could be further improved, since an anatomically accurate model is required to simulate the complexity of these tissues.

The accurate anatomy of the ACL is essential for understanding its function and biomechanical behavior. With the in-depth anatomical studies, some researchers found that the ACL is a complete bundle consisting of a central bundle and fan-shaped fibers [26,27,28], and the femoral insertion was close to an oval shape. In contrast, the tibial insertion was approximately an irregular oval or “C”-shaped. In addition, Zhang et al. reported that a modified ACL reconstruction technique for the tibial and femoral tunnel resulted in better postoperative function performance compared to conventional ACL reconstruction [29]. The morphology and insertion of the ligament in the knee joint model greatly influence the force characteristics of the ligament. Future studies could improve the similarity of the geometry of the ligaments used in this study to the anatomical morphology of human ligaments. Therefore, it is essential to build a knee model with a fine morphology of the ACL.

The purpose of this study was to (1) develop a finite element model of the knee joint that conforms to the actual anatomical morphology of the ACL and validate it through cadaveric experiments; (2) to analyze the stress distribution within the ACL under different loading conditions to provide a basis for the further investigation of the risk factors of ACL injury.

## 2. Materials and Methods

### 2.1. Specimen Preparation

A right fresh-frozen human cadaveric knee from a male donor (32 years old) was used in the study. The research protocol was approved by the Ethics Committee of Peking University Third Hospital (IRB00006761-M2019056), with a donation to the Department of Anatomy and Histoembryology. Examinations were performed to ensure that this specimen had no anatomical abnormalities, no history of knee trauma and no significant restriction of joint mobility.

Cut specimen: the femur and tibia were kept 20 cm from the knee joint. As the distal fibula was removed, the proximal tibiofibular joint stability may be affected. So, the proximal end was fixed with 3 cm diameter cortical bone screws, and all soft tissues above 10 cm from the knee joint were removed. The knee specimen was kept moist with 0.9% physiological saline throughout the experiment. The ends of the femur and tibia were fixed to custom-made aluminum clamps.

### 2.2. Generation of the Finite Element Model of the ACL

A computed tomography (CT) scan was first performed to obtain the bone tissue structures using CT equipment (GE Healthware, Wauwatosa, WI, USA) with a layer thickness of 0.6 mm, a layer spacing of 0.6 mm and a resolution of 512 × 512. The magnetic resonance data were then obtained from the cadaveric knee specimen by a 3-T MR scanner (voxel size 0.7 mm × 0.6 mm × 0.7 mm, resolution 256 × 256, medic3d sag fs) with simultaneous scans in the sagittal, coronal and axial positions with 144 layers, and the scan position was in the knee extension.

The bone, ligaments and cartilage were segmented with CT bone segmentation operation in MIMICS software (Materialise, Leuven, Belgium). The ligaments, cartilage and meniscus were segmented from magnetic resonance imaging (MRI). The segmentation was first performed using the software’s built-in segmentation method and then adjusted under the guidance of an experienced orthopaedist (Z.L.). All the segmented tissues were reconstructed into a 3D geometric model in MIMICS software and exported to a file in stereolithography (STL) format. The completed models were imported into Geomagic software for more detailed geometric model processing, including adjusting and reconstructing the poor-quality surfaces in the model and smoothing the surface pieces of the model so that the model could retain the complex anatomical features of the original structure while having a good enough smoothness to lay the foundation for the later meshing. Then, the contour lines of the model were extracted, the raster was constructed, the surface was fitted and the surface sheet model was finally constructed and exported as the Initial Graphics Exchange Specification (IGES) format for the subsequent meshing. The surface models in IGES format were imported into Hypermesh software for meshing. Tetrahedral elements were generated with an element size of 1 mm for the ligament and cartilage tissues and 3 mm for the bone tissues.

The established 11 parts, including the femur, tibia, fibula, anterior cruciate ligament (ACL), posterior cruciate ligament (PCL), medial collateral ligament (MCL), lateral collateral ligament (LCL), medial meniscus, lateral meniscus, tibial cartilage and femoral cartilage, were imported into ABAQUS software (Dassault Systemes Simulia Corp., Johnston, RI, USA) for material property assignment and related boundary condition and load definition. In this study, bone was defined as a linear elastic material, all ligaments were defined as hyperelastic materials and the Neo-Hooker model was chosen. The material properties of each tissue of the knee joint are shown in Table 1 [30,31,32,33].

The fibula head and the posterior and lateral tibial epicondyle form the proximal tibiofibular joint, which hardly undergoes any sliding motion, so the tibia and fibula are defined as a fixed contact. The ACL, PCL, MCL and LCL insertion surfaces on the femur and tibia were attached to the bone and treated as a rigid surface. The contact during the calculation was defined as a hard contact without friction, limited slip or penetration, containing the contact between the femur and the ACL, the tibia and the ACL, the femur and the MCL, the tibia and the MCL, the femur and the tibia and the ACL and the PCL, and the contact simulation was performed using the general contact algorithm in ABAQUS software. The femur was fixed in all translational and rotational degrees of freedom, and the distal tibia was constrained in the flexion and extension degrees of freedom. The boundary conditions were set to be consistent with those implemented in the biomechanical experiment of the same knee specimen.

### 2.3. Simulations

Under the boundary conditions described above, two analyses were performed to mimic the experimental configuration, with the femur fixed and the tibia free to move in five degrees of freedom (DOF). First, simulations of the three loading conditions on the intact knee were performed, including: (1) an anterior tibia force of 134 N was applied to the proximal end of the tibia, and the anterior displacement of the tibia and the stress of the ACL were calculated; (2) a 5 Nm external tibial torque was applied to the proximal end of the tibia to calculate the rotation angle of the tibia and the stress of the ACL; (3) a 5 Nm internal tibial torque was applied to the proximal end of the tibia to calculate the rotation angle of the tibia and the stress of the ACL. Then, remove the ACL and repeat these three loading conditions. In order to test the accuracy of the model analysis, the loading conditions were set to be consistent with the loading protocol implemented in the biomechanical experiment of the same knee specimen.

### 2.4. Biomechanical Test for Finite Element Model Validation

**Figure 1:**
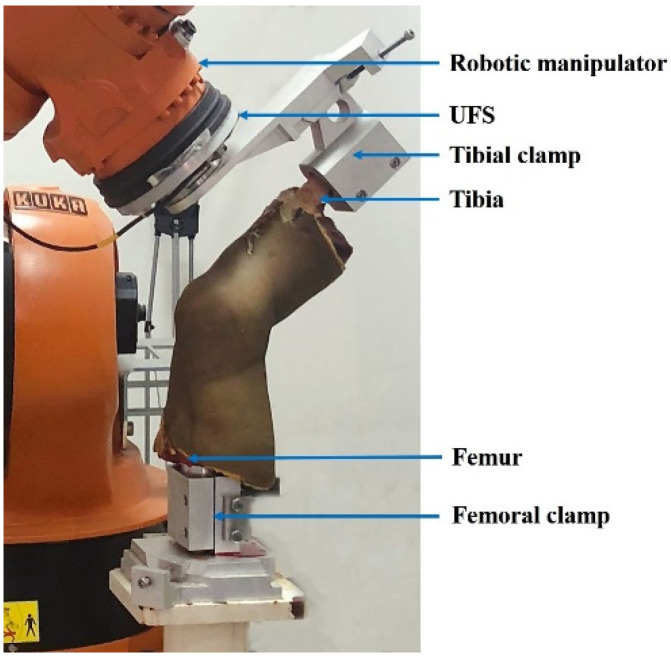
Human cadaveric knee model tested with robotic universal force/moment sensor (UFS) testing system.

After the CT and MR scans, biomechanical tests of the same knee specimen were performed using the robotic universal force/moment sensor (UFS) testing system to validate the knee model. The specimen was mounted in the UFS testing system, as in previous studies [34,35]. The femoral side was rigidly fixed to the base, and the tibia was fixed to the sensor of the robot (Figure 1). The robot (KUKA Robots, Augsburg, Germany) allows for knee movement at 6 DOF. The UFS can measure three forces and three moments in a Cartesian coordinate system, and the resolution is 0.25 N for forces and less than 0.075 Nm for moments.

The following external loading conditions [34,36] were applied to the tibia at the knee extension, and the corresponding knee kinematic data were recorded: (1) a 134 N anterior tibial load was given, and the anterior tibial displacement was recorded; (2) a 5 Nm external tibial torque was given, and the external rotation angle was recorded; (3) a 5 Nm internal tibial torque was given, and the internal rotation angle was recorded. The loading protocol was used to simulate the anterior drawer test or the pivot shift test in clinical examinations. The knee kinematic responses of the intact knee under the three loading conditions were recorded. Then, the ACL was removed under arthroscopy, re-fixing the specimen in the UFS system, repeating the measurement of the above loading conditions and recording the data. The experimental data were compared with the results of the finite element simulation analysis.

## 3. Results

### 3.1. Model Establishment

**Figure 2:**
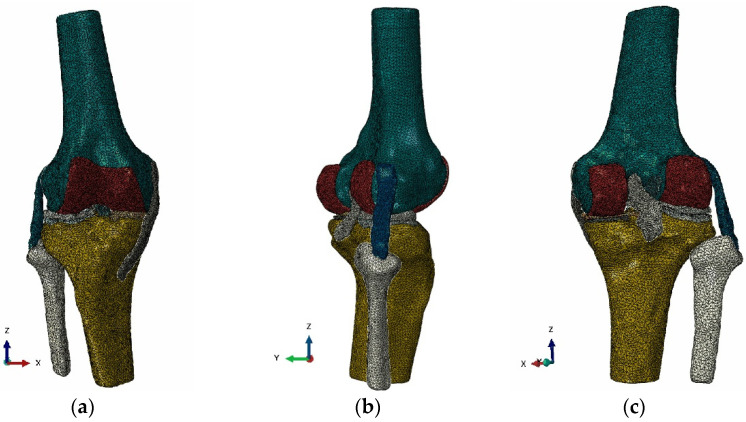
Finite element model of the right knee joint: (**a**) Front view; (**b**) Side view; (**c**) Rear view.

**Figure 3:**
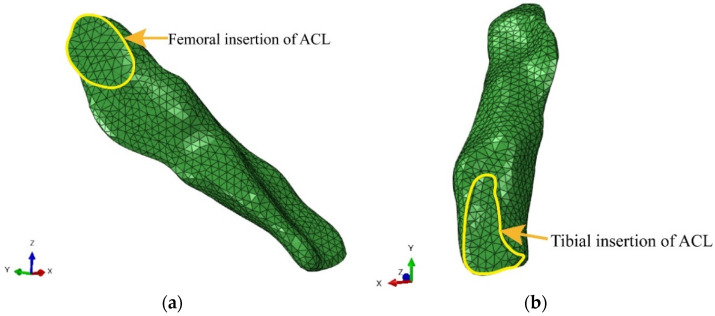
The morphology of the femoral and tibial insertions in the ACL: (**a**) The outline of the red mark indicates the femoral insertion of the ACL; (**b**) The outline of the red mark indicates the tibial insertion of the ACL.

A three-dimensional finite element model involving the distal segment of the femur, the proximal segment of the tibia and fibula, the ACL, the PCL, the MCL, the LCL, the medial and lateral meniscus, the tibial cartilage and the femoral cartilage was established using the ABAQUS software (Figure 2). The model contained 137,405 nodes and 430,962 elements. The overall shape and the femoral and tibial insertions of the ACL were finely segmented in this study. The femoral insertion of the ACL was an oval shape, and the tibial insertion of the ACL was long “C”-shaped. The model vividly reflected the ACL geometry (Figure 3) and could accurately calculate the ligament stress.

### 3.2. Validation of the Finite Element Model

**Figure 4:**
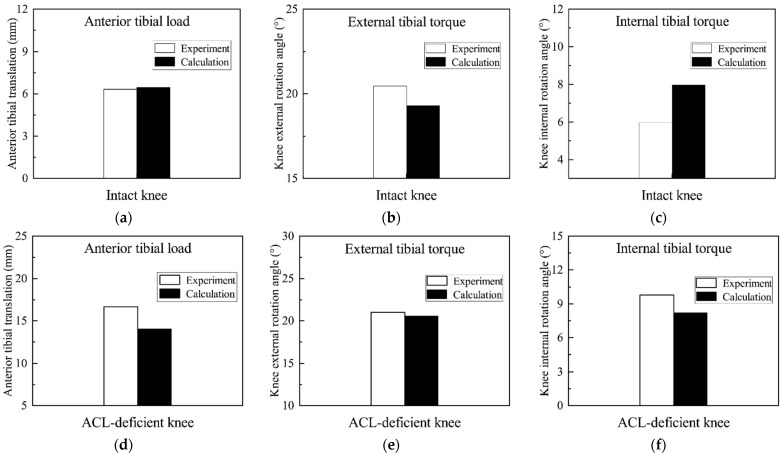
Comparison of the specimen experimental and finite element model results: (**a**) Anterior tibial translation under anterior tibial load in the intact knee; (**b**) Knee external rotation angle under external tibial torque in the intact knee; (**c**) Knee internal rotation angle under internal tibial torque in the intact knee; (**d**) Anterior tibial translation under anterior tibial load in the ACL-deficient knee; (**e**) Knee external rotation angle under external tibial torque in the ACL-deficient knee; (**f**) Knee internal rotation angle under internal tibial torque in the ACL-deficient knee.

Figure 4 shows the comparison of the knee kinematics between the experimental test results and the model-based calculations for the intact knee and the ACL-deficient knee, respectively. For the intact knee condition, the results of the specimen experiments showed that the anterior tibial translation was 6.32 mm under the anterior tibial load, the external rotation angle of the tibia was 20.5° under an external rotation torque and the internal rotation angle of the tibia was 6.0° under an internal rotation torque. Under the same loading conditions as the specimen, the results of the simulations were similar to those of the specimen. The model-based calculations showed that the anterior tibial translation was 6.44 mm under the anterior tibial load, the external rotation angle of the tibia was 19.3° under an external rotation torque and the internal rotation angle of the tibia was 7.96° under an internal rotation torque.

For the ACL-deficient knee condition, the results of the specimen experiments showed that the anterior tibial translation was 16.68 mm under the anterior tibial load, the external rotation angle of the tibia was 21.0° under an external rotation torque and the internal rotation angle of the tibia was 9.8° under an internal rotation torque. Under the same loading conditions as the specimen, the model-based results were similar to those of the specimen in the ACL-deficient knee. The model-based results showed that the anterior tibial translation was 14.05 mm under the anterior tibial load, the external rotation angle of the tibia was 20.6° under an external rotation torque and the internal rotation angle of the tibia was 8.2° under an internal rotation torque.

### 3.3. ACL Stress under Different Loading Conditions

**Figure 5:**
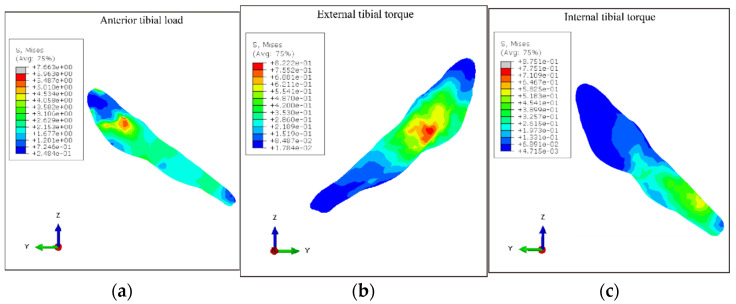
Stress distribution of the ACL: (**a**) ACL stress distribution under anterior tibial load; (**b**) ACL stress distribution under external tibial torque; (**c**) ACL stress distribution under internal tibial torque.

The stress characteristics of the ACL under different loading conditions are shown in Figure 5. Under the anterior tibial load, the highest maximal principal stresses were localized on the posterior region of the femoral insertion of the ACL, with a maximum of 14.884 MPa near the femoral insertion site. Under the external tibial torque, the highest maximal principal stresses were mainly concentrated on the mid-substance of the ACL, with a maximum of 0.815 MPa. Under internal tibial torque, the highest maximal principal stresses mostly took place near the tibial insertion site, with a maximum of 0.933 MPa.

## 4. Discussion

This study developed human knee joint finite element models based on accurate geometrical entities in order to investigate their mechanical behavior. The ligament tissues were finely segmented under a sports medicine physician’s professional guidance, especially in the ligaments attached to the bone. The ACL femoral insertion in this model was oval-shaped, and the tibial insertion of the ACL was long “C”-shaped. Previous anatomical studies have found that the tibial ACL midportion and the tibial “C”-shaped insertion are flat and resemble a “ribbon” [28]. A novel ACL reconstruction technique with a rounded rectangle-shaped tibial tunnel and an oval-shaped femoral tunnel has been developed to mimic the anatomical orientation and shape of the tibial and femoral footprints [29]. The morphology of the ACL is fundamental for its biomechanical behavior. A biomechanical cadaveric study reported that the graft position in ACL reconstruction greatly influenced biomechanical performance [37], indicating the importance of matching native ACL footprints as close as possible. Based on the fine ligament structure in the current model, the force characteristics of the ligament during motion could be more accurately explored. The accurate anatomical structure of the model can lay the foundation for the subsequent simulation of ligament force.

The same cadaveric specimen was also experimentally studied in this study. The model was validated by comparing the kinematic changes measured by the experiment of the same knee specimen under identical loading conditions with those calculated by the model. The results in this study demonstrated that the model calculations in the intact and ACL-deficient knee were similar to the experimental results of the specimen. The experimental results showed an increase in anterior tibial translation and an increase in external and internal rotation angles in the ACL-deficient knee compared to the intact knee. In the model calculation results, the anterior tibial translation, external tibial rotation angle and internal tibial rotation angle also showed an increasing trend when the model without the ACL was subjected to the same load of the anterior tibial load, external tibial torque and internal tibial torque compared with the model with the intact ACL. Therefore, not only were the calculated results of the model similar to the experimental results, but the trend of the calculated results of the model after ACL fracture was also consistent with the experimentally measured trend.

The results of the study showed that, in the knee extension position, the peak ACL stresses were larger when the tibia was under anterior tibia load compared with the ACL stresses under the conditions of external and internal tibial torque. The results indicated that the dangerous load that causes ACL injury is mainly the anterior tibial load. The tibial rotation has a smaller stress load on the ACL and contributes less to its injury. In addition, the location of peak stresses on the ACL differed between load types: under anterior tibial load, peak ACL stresses were located near the femoral insertion site, i.e., the upper part of the body of the ligament; under external tibia torque, peak ACL stresses were mainly located in the middle region of the body of the ligament; under internal tibia torque, peak ACL stresses were found at the tibial insertion site of the ACL, i.e., the lower part of the body of the ligament. The result of this study was consistent with the previous literature. Xie et al. also investigated the ACL stress distribution when an anterior tibial load at knee extension was applied [22]. They also found that the focus of the ACL stress was near the femoral insertion under anterior tibial loading. In addition, the result was consistent with the clinical observations. Previous studies of clinical data on ACL injuries had shown that acute ACL ruptures occur most commonly in the upper part, with an incidence of 72.2%, followed by the central rupture rate of 26.4%, and ACL rupture sites were rarely seen in the lower end of the ligament [38]. Therefore, combined with the location of maximum force and clinical data showing the rupture location, the most dangerous load during ACL injury may be the anterior tibial load.

After this validation, the model was applied to investigate the role of the ACL in resisting rotational moments. The ACL injury mechanism is still controversial in sports medicine, and the current biomechanical evidence is inconclusive [39]. A recent study recreated the tibia-femoral position near the time of an ACL injury based on bone bruises and found that patients with ACL injuries with bone bruises only on the lateral side of the knee were in knee flexion, valgus and tibial external rotation during the injury [40]. The results suggest that knee valgus combined with knee external rotation motion in knee flexion might be the primary mechanism leading to an ACL injury in patients with bone bruises on the lateral side of the knee. However, other researchers suggested that internal rotation causes ACL ruptures, while external rotation occurs after the rupture [41]. Bone bruise analysis can only provide results and cannot determine the time series, while finite element analysis can offer a new perspective for injury mechanism exploration and verification. Like the discussion in the previous paragraph, this study found that the location of peak stress under external tibial torque was more common for ACL rupture than under internal tibial torque. In contrast, peak forces under internal tibial torque mainly occur near the region of the tibial insertion, which is rarely seen in clinical practice. Thus, it could be inferred that ACL injuries are more frequently loaded by external tibial than internal tibial torque.

With the FE model developed in the current study, studies of the ACL injury mechanism could be explored in the future. The finite element method can simulate the dynamic analysis and investigate the ligament stress during movement by inputting the movement parameters as loading conditions into the finite element model. Exploring the stress characteristics of the ACL and further determining the key factors affecting the ACL stress will reveal the risk factors for ACL injury. The validation and optimization of ACL reconstruction, such as the tunnel position or orientation, are also needed [42]. Since this model contains the cartilage and meniscus, the effects of ACL injury or reconstruction on the cartilage and meniscus could be simulated by the model in future studies [43,44]. The model can be applied to the mechanism of a variety of clinical and research problems.

The knee model contains the patellofemoral, tibiofemoral and upper tibiofemoral joints. Since this study focused on the influence of the loading conditions of the tibiofemoral joint on the ACL force, to appropriately simplify the model, the model established in this study did not include the patella and its associated ligamentous tissues and did not consider the influence of the patellar tendon, quadriceps, hamstrings and other structures on the ACL stress. The calculation results might differ from the actual situation to some extent. Future studies should try to maintain the integrity of the knee joint model and investigate its effect on ligament stress. In addition, to maintain the consistency of the specimens used for model building and experimental validation, only one specimen was made for validation. Since soft tissue is viscoelastic and time-dependent, there is a possibility that the loading condition relaxed the ligaments and other soft tissues, and, thus, testing under a second or third loading condition could not be representative of what happens under a non-stressed knee. The validation of the model with only one sample is another limitation of the current study. Future studies should experimentally validate with larger sample sizes and ensure that a new specimen is used for each loading condition.

## 5. Conclusions

In summary, this study established a finite element model of the knee, validated the model by experiments on the same cadaveric knee specimen and analyzed the ACL stress characteristics under different load conditions based on the model. The anatomical features of the tissues in the model had a high geometric similarity, and the simulation results of the established knee joint finite element model are in good agreement with the experimental results. The FE model presented in this study could be a valuable tool in investigating the ACL injury mechanism and optimizing ACL reconstruction surgery.

Compared with the ACL stress under tibial external and internal rotation load, the ACL stresses were larger when the anterior tibial load was applied. The highest maximal principal stresses were localized on the femoral insertion of the ACL under the anterior tibial load, while under the external and internal rotation load, the highest maximal principal stresses mostly took place near the mid-substance of the ACL and the tibial insertion site, respectively. Combined with the location of maximum force and acute ACL rupture in clinical practice, the most dangerous load during ACL injury may be the anterior tibial load. ACL injuries were more frequently loaded by external tibial torque than internal tibial torque.

## Figures and Tables

**Table 1:** Material parameters of the model.

Ligament	Material Parameters
Bone tissues	E = 17,000 MPa, ν = 0.36
Cartilage	E = 20 MPa, ν = 0.45
Meniscus	E = 59 MPa, ν = 0.49
Anterior cruciate ligament	C_1_ = 1.95, D = 0.00683, ν = 0.49
Posterior cruciate ligament	C_1_ = 3.25, D = 0.0041
Medial collateral ligament	C_1_ = 1.44, D = 0.00126
Lateral collateral ligament	C_1_ = 1.44, D = 0.00126

E, elastic modulus; ν, Poisson’s ratio; C_1_, the Neo-Hooker constant; 1/D, the Bulk Modulus.

## Data Availability

The data presented in this study are available on request from the corresponding author.
